# Children reporting rescuing other children drowning in rural Bangladesh: a descriptive study

**DOI:** 10.1136/injuryprev-2013-041015

**Published:** 2014-03-31

**Authors:** Tom Stefan Mecrow, Aminur Rahman, Michael Linnan, Justin Scarr, Saidur Rahman Mashreky, Abu Talab, A K M Fazlur Rahman

**Affiliations:** 1 International Drowning Research Centre—Bangladesh (IDRC-B), Dhaka, Bangladesh; 2 The Alliance for Safe Children (TASC), Atlanta, USA; 3 Royal Life Saving Society Australia (RLSSA), Sydney, Australia; 4 Centre for Injury Prevention Research, Bangladesh (CIPRB), Dhaka, Bangladesh

## Abstract

**Background:**

SwimSafe, a basic swimming and safer rescue curriculum, has been taught to large numbers of Bangladeshi children since 2006. This study examines the frequency and characteristics of rescues reported by children who graduated from SwimSafe and compares them with age-matched and sex-matched children who did not participate in SwimSafe.

**Methods:**

Interviews were conducted during the swimming season in Raiganj, Bangladesh. Data were collected from 3890 SwimSafe graduates aged 6–14. Two age-matched and sex-matched controls were selected; one who had learned to swim naturally, the other who had not learned to swim.

**Results:**

188 rescues were reported by the three groups. The 12–14-year age groups reported the highest monthly rate of rescues (SwimSafe 10.5/100 000 (95% CI 3.4 to 24.5), natural swimmers 8.5/100 000 (95% CI 2.2 to 21.2)) and annual rate of rescue reported (SwimSafe 25.4/100 000 (95% CI 13.2 to 43.9), natural swimmers 35.4/100 000 (20.8 to 56.2)). Reported rescue numbers among both swimming groups was similar. Mean victim age was 4.1 years and 92.5% were under 7 years. All victims were younger than their rescuer (mean 5.9 years less). Most rescues (73.7%) took place in ponds or ditches with most (86.6%) within 10 m of the bank. Most victims had entered the water to bathe (53.8%). A large majority of reported rescues (90.9%) were conducted with the rescuer in the water, half requiring the rescuer to swim.

**Conclusions:**

Children report frequent drowning rescues of younger children in rural Bangladesh. Most reported are contact rescues with the rescuer in the water. Formal training for in-water rescue techniques may be needed to reduce the risk to the child rescuer.

## Introduction

Globally the burden of drowning is high and especially so in low and middle income countries (LMICs).^1^ This is true in Bangladesh where the 2003 Bangladesh Health and Injury Survey (BHIS) found drowning to be a leading cause of death in children in Bangladesh with many reports of children drowning attempting to rescue a peer who was drowning.^2^ The high drowning rates found relate to widespread daily exposure of children to water bodies in rural areas.

The SwimSafe basic swimming curriculum was created in 2005 by the Centre for Injury Prevention, Bangladesh (CIPRB), The Alliance for Safe Children (TASC) and Royal Life Saving Society Australia (RLSSA) in response to the high drowning rates found in the BHIS survey.^3^ SwimSafe is a basic swimming, non-contact rescue and water safety programme developed in Bangladesh, Thailand and Vietnam for the low-resource environments of LMICs. In Bangladesh, the SwimSafe programme has been taught to over 250 000 children 4–16 years old since 2006. SwimSafe has been shown to be effective in preventing drowning.^4^ In addition to swimming, SwimSafe teaches children safer rescue using non-contact, land-based reach and throw techniques. Children are not taught contact rescue techniques and are taught only to enter the water as a last resort.

This exploratory study gathered information on the frequency and characteristics of rescues reported by children who graduated from SwimSafe as well as for age-matched and sex-matched peers who had no contact with SwimSafe.

## Methods

Trained interviewers used a structured and pretested questionnaire to conduct interviews at two different times in Raiganj, Bangladesh. The first was March–April 2011, when frequent swimming begins as ponds fill and the weather becomes warm. The second was September–October 2011, late in the swimming season when swimming and water play is high.

Raiganj is a landlocked rural district with a population of 3 097 450.^5^ Most homes have small ponds nearby, and most villages have several large ponds. Many small and large rivers intersect the district.

To explore the frequency and method of rescues in the child population, a target number of 4000 SwimSafe graduates were randomly selected from a list of approximately 200 000 graduates of the rural SwimSafe programme. Two controls were randomly selected from a household registration list in the same village block of approximately 1200 homes for each graduate, matched for age and sex. One was a child who had learned to swim naturally without exposure to the SwimSafe programme. The other was a child who had not yet learned to swim as reported by the child's parents and who had not had exposure to the SwimSafe programme.

SwimSafe graduates were categorised into three age groups: 6–8 years (1322), 9–11 years (2096) and 12–14 years (472). The sample age distribution reflected the age distribution of the SwimSafe programme with the largest number in the 9–11-year age group. The 6-year lower age boundary was chosen to ensure interviews could be successfully completed with the child. A total of 3890 SwimSafe graduates, 3924 natural swimmers and 3903 non-swimmers had completed interviews.

The study was approved by the CIPRB ethical review committee, and consent was obtained from parents prior to face-to-face interviews with the children. Children were told a rescue had occurred if a victim was in water deeper than his/her height, needed assistance and was unable to exit the water until he/she intervened. A child was then asked, “Have you ever rescued a friend or other person who was in trouble in the water?” Responses were recorded for the number of rescues ever done. The question was asked again limited to the previous 1 month to establish an incident time period. Information was collected on number of rescues reported, approximate date of rescue, rescuer and victim characteristics of each rescue, and place, type, circumstances and outcome of each rescue. Victim characteristics were obtained by interviews with victim parents once the identity and residence of the victim was determined in the rescuer interview. Reported rescue rates were calculated based on the rescue numbers reported and the number of children in each group. Data entry and analysis was done using SPSS V.20. Pearson's χ^2^ test was used to compare categorical responses.

## Results

The three groups reported a total of 188 rescues ever conducted: 95 SwimSafe, 91 natural swimmers and 2 for the non-swimmers. Two rescues were reported from the 3943 non-swimmers, which is too few to calculate meaningful rates and proportions. Results follow for the SwimSafe and natural swimming groups only.

Of the 186 ever-conducted rescues reported by the two swimming groups, 48 were reported in the 1 month preceding the interview, 121 were reported 2–12 months preceding the interview, 12 were reported 13–24 months preceding the interview and 5 were reported in the 25–36 months preceding the interview. All rescues took place during the day with most (53.8%) reported during lunchtime between 12:00 and 13:59. Nine out of ten (91.4%) took place between 10:00 and 15:59.

The 12–14-year age group in both swimming groups had the highest monthly and annual reported rescue rate. There was no statistical difference in any age group between reported SwimSafe and natural swimming rescue numbers and rates in either 1 month or 1 year. There was also no statistical difference between the number of rescues conducted by males and females in both the SwimSafe (χ^2^=1.27, p=0.26) and natural swimming (χ^2^=0.98, p=0.32) groups (table 1).

**Table 1:** Reported rescue rates by swimming group, age group and time period

Time period	Age (years)	SwimSafe (SS)		Natural swimmer (NS)		χ^2^ SS vs NS	p Value
		Number	Rate/1000 in group (95% CI)	Number	Rate/1000 in group (95% CI)		
Previous month	6–8	6	4.5 (1.6 to 9.8)	6	4.2 (1.5 to 9.3)	0.01	0.92
	9–11	18	8.5 (5.0 to 13.5)	9	4.3 (2.0 to 8.3)	2.82	0.09
	12–14	5	10.5 (3.4 to 24.5)	4	8.5 (2.2 to 21.2)	0.13	0.72
	6–14	29	7.4 (4.9 to 10.6)	19	4.8 (2.9 to 7.5)	2.18	0.13
Previous year	6–8	17	12.8 (7.5 to 20.5)	15	10.7 (6.0 to 17.6)	0.26	0.6
	9–11	61	29.1 (22.3 to 37.2)	47	22.9 (16.9 to 30.3)	1.55	0.21
	12–14	12	25.4 (13.2 to 43.9)	17	35.4 (20.8 to 56.2)	0.81	0.36
	6–14	90	23.1 (18.6 to 28.3)	79	20.1 (15.9 to 25.0)	0.83	0.36

Total of 48 rescues in 1 month and 169 in 1 year; 18 rescues excluded as beyond last 365 days.

Victim mean age was 4.1 years, and nearly all victims (92.5%) were less than 7 years old. All victims were younger than their rescuer, with a mean of 5.9 years younger, and there were similar proportions of male and female victims (data not shown). There was no significant difference in numbers and ages of victims reported rescued by SwimSafe or natural swimmers (table 2).

**Table 2:** Age of victim and swimming ability of rescuer at time of rescue

Rescuer swimming ability	Age of victims (years)									Total
	1	2	3	4	5	6	7	8	9	
SwimSafe	2	17	22	25	12	10	3	3	1	95
Natural swimmer	2	15	13	26	17	11	5	1	1	91

*χ^2^=0.17, p=0.678 for difference between 95 total SwimSafe children and 91 total naturally swimming children.

Almost three-quarters (73.7%) of rescues took place in small bodies of water (ponds or ditches) and about 9 of 10 (87.6%) took place within 10 m of the bank, with almost half (46.9%) within 5 m of the bank in ponds and ditches and rivers, the most common places of rescue (table 3).

**Table 3:** Place and distance of reported rescues conducted

Place (% among places)	≤5 m (n, %)	6–10 m (n, %)	≥11 m (n, %)
Pond, 52.7%	41, 41.8	38, 38.8	19, 19.4
Ditch*, 21.0%	13, 33.3	24, 61.5	2, 5.1
River, 17.2%	21, 65.6	10, 31.2	1, 3.1
Canal, 8.1%	6, 40.0	8, 53.3	1, 6.7
Lake, 0.5%	0, 0	1, 100	0, 0
Other, 0.5%	0, 0	1, 100	0, 0

*A low area close to the home that is normally dry that fills up with large amounts of water in the rainy season.

More than half (53.8%) the victims entered the water for bathing. A quarter of victims (24.7%) were playing in the water when they got into difficulty. Fifteen per cent did not intentionally enter the water but fell into it. Only one child (0.5%) entered for the express purpose of swimming. Nine out of ten victims (90.3%) were identified as needing rescue because of actions of others—rescuers saw they were struggling, saw them go under and stay under, or someone other than the victim called for help. Victims themselves uncommonly called for help (7.5%) (table 4).

**Table 4:** Reported reason for victim entry into water and how victim was identified

Reason entry in water (% reason for entry)	Struggling (n, %)	Under water (n, %)	Other called (n, %)	Victim called (n, %)	Unknown (n, %)
Bathing, 53.8%	65, 65.0	23, 23.0	5, 5.0	0, 0.0	0, 0.0
Playing, 24.7%	26, 56.5	4, 8.7	7, 15.2	5, 10.9	4, 8.7
Fell in, 15.6%	22, 75.9	5, 17.2	1, 3.4	1, 3.4	0, 0.0
Pick something up, 3.8%	3, 42.9	0, 0.0	3, 42.9	1, 14.3	0, 0.0
Swimming, 0.5%	0, 0.0	0, 0.0	1, 100	0, 0.0	0, 0.0
Don't know, 1.6%	1, 33.6	2, 66.7	0, 0.0	0, 0.0	0, 0.0

In a large majority of cases, the rescuer entered the water to conduct the rescue in both groups (SwimSafe graduate (SS) 82.1%, Natural Swimmer (NS) 82.4%). Almost one-tenth of rescuers (SS 8.4%, NS 8.8%) were already in the water when they reported seeing or hearing the victim was in trouble. In almost all in-water rescues (99.1%), the rescuer made direct contact with the victim for both groups (data not shown). Land-based rescues (reach and throw) were <10% of all rescues (SS 9.5%, 8.8%).

Swimming rescues where the rescuer had to swim to reach the victim accounted for about half of all in-water rescues (data not shown). There was no difference in swimming rescue in SwimSafe graduates (47.4%) compared with natural swimmers (52.7%) (χ^2^=0.54, p=0.46). In the remainder of in-water rescues, the rescuer was able to stand up in the water and reach the victim without swimming (table 5).

**Table 5:** Reported method of rescue

	Water rescue		Land rescue			Total
	Entered water	Already in water	Reach by pole	Reach by hand	Throw rescue	
SwimSafe	78 (82.1)	8 (8.4)	4 (4.2)	4 (4.2)	1 (1.1)	95 (100)
Natural swimmer	75 (82.4)	8 (8.8)	4 (4.4)	4 (4.4)	0 (0.0)	91 (100)

Almost all rescues were successful (99.5%) with one fatal drowning (data not shown). Most victims discontinued swimming/playing or sought assistance (88.2%), and 8.1% continued swimming or playing. In four cases (2.3%), victims were unconscious but breathing.

## Discussion

This exploratory study was done to determine the frequency and patterns of self-reported rescues in three sex-matched and age-matched groups of Bangladeshi children 6–14 years old: (1) those who graduated from SwimSafe, a basic swimming and safer rescue programme; (2) those who were not SwimSafe graduates but learned to swim on their own (natural swimmers); and (3) those who were not SwimSafe graduates and had not yet learned to swim. Slightly less (11 717) than the target of 12 000 children in all three groups responded to the survey over both time periods. Children in group 3 (non-SwimSafe graduates and non-swimmers) only reported two rescues, which was insufficient to establish a meaningful rescue rate. For the other two groups, findings showed large numbers of rescues reported by both SwimSafe children and natural swimmers. Most reported rescues for both groups took place in ponds and ditches with rescuers entering the water. The methods and frequencies of rescue activities did not differ between SwimSafe graduates and natural swimmers.

The observation of only two rescues conducted by the non-swimming group and the similar rescue rates between the two groups able to swim suggests the ability to swim or confidence in the water environment was the common enabling factor for rescues to be attempted.

In this study, successful rescues were reported by young children at high rates in both 1-month recall and annual recall periods. The reported rates are much higher than those seen with bystander rescues in high-income countries (Justin Scarr, personal communication, Drowning Commissioner, International Life Saving Federation and CEO, Royal Life Saving Society, Australia, August 2013). The timing of the samples likely explains the large difference between the 1-month rate when annualised and the rate measured over 1 year. Times were chosen to coincide with the beginning and peak of the period when children play or swim in water. One-month rates during this 5-month period would be expected to be higher than those during the rest of the year when temperatures are cold and pond levels are low. The BHIS national survey showed drowning rates are markedly affected by seasonality. Logically, rescue rates would follow this pattern since most rescues were for bathing, which is less seasonal than swimming but also decreases in cold weather.

In rural Bangladesh, the necessarily frequent use of ponds for essential daily tasks, coupled with large family sizes and busy morning routines for mothers, means that supervision of very young children in and around water is often delegated to an older sibling. Many of the findings likely result from this cultural norm of sibling supervision. These include (1) most reported drowning victims entered the water without adult supervision; (2) in all reported rescue events, the rescuer was a child and an adult did not provide assistance nor was involved in any other way; (3) for children able to swim and reporting rescuing other children, victims were very young children (mean age 4.1 years) with an age difference between rescuer and reported victim of about 6 years (mean 5.9 years); and (4) most rescues (53.8%) occurred from 12:00 to 13:59 h, a time where most rural mothers are busiest with household chores.

The lack of a difference in frequency of reported rescues between SwimSafe graduates and children who learned swimming from their peers or parents may also be related to sibling supervision. In each group, very young children often got into trouble while bathing, requiring the supervising older sibling to conduct a rescue.

Other cultural characteristics of rural Bangladesh may also have resulted in another finding. Despite being taught safer land-based rescue techniques and to enter the water only as a last resort, SwimSafe children were equally likely to enter water to conduct a rescue. Rescuers reported a lack of sticks and branches at the pond or ditch to allow a reach rescue. In rural Bangladesh, sticks or branches are rarely available having been collected for firewood. Their absence likely left water entry the only available option.

Despite the surveys being timed to occur at periods where recreational water exposure (swimming and play in water) was maximal, only one in four (25.2%) reported rescues occurred when a victim was engaged in recreation (swimming 0.5% or playing 24.7%). Almost three out of four reported rescues (73.2%) occurred when a very young child entered the water for bathing (53.8%), fell in unintentionally (15.6%) or entered to retrieve something that had fallen in (15.6%). In large part due to the cultural norm of sibling supervision of very young children, bathing appears to be among the highest risk exposures to water for young children in rural Bangladesh.

The relationship between adult supervision and an increased risk of drowning has been shown to influence other types of unintentional child injury in high income countries (HICs) and LMICs.^6^ ^7^ In HICs, professional services are often used to mitigate this risk (eg, provided by a teacher or lifeguard). Importantly, awareness needs to be raised in the community regarding the risk associated with young children bathing in bodies of water without adequate supervision. Further research is underway to characterise community attitudes regarding the age necessary for an older sibling to effectively supervise a young child around water.

The study suggests in-water rescue techniques should be added to the already included land-based techniques in the SwimSafe programme. Nine out of ten rescues in this study (90.2%) were in-water rescues where the child rescuer had contact with the child victim. Rescue equipment and supervising adults were not present, and the victim was either alone or accompanied by peers or a sibling. Cultural norms such as sibling supervision are resistant to change and doing so will likely require a substantial length of time.

In HIC settings, adult drowning while rescuing children has been described as ‘aquatic victim instead of rescuer syndrome’ (AVIR).^8^ This has been one factor in stimulating development of rescue techniques, policies and education programmes aimed at reducing the risk to the rescuer, especially when there is potential for direct contact with the victim in the water. Safety legislation in HIC settings usually requires safety equipment and professional supervision in regularly used public swimming areas. This has markedly lessened the need for a bystander rescuer to enter the water to conduct a contact rescue. Learning rescue techniques such as contact towing used to be common in basic lifesaving courses. Now many HIC lifesaving organisations restrict teaching of contact tows to more advanced learners.^9^ As a result, children in HICs are usually only taught land-based rescue techniques in entry-level courses.

The drowning environment in Bangladesh is very different from that in HIC settings. In this environment, the study found that children conduct in-water rescues that involve contact even when having received training in safer land-based techniques. This apparently results from both the nature of the drowning environment and a lack of reach extension devices and other safety equipment rather than an inability to learn the safer land-based technique.

Given that in-water rescue is the most commonly used technique by children in rural Bangladesh, risks associated with it should be mitigated. Teaching this may be viewed as having the potential to increase risk to the child rescuer. However, the finding that it is already the norm for conducting rescues argues for a risk reduction approach. Such techniques may be quite simple. An example would be for the rescuer to take off their t-shirt (the usual clothing worn by children) and extend that to the victim, avoiding direct contact. Furthermore, only about half of in-water rescues required swimming to make contact with the victim. Most rescues were conducted close to the bank where water is usually shallow and the older child rescuer was tall enough to reach the younger victim without swimming. Logically, these would entail less risk to the rescuer due to the ability to stand as well as the large difference in physical development found between children in early childhood and later childhood, which was the difference seen between the rescuer and the victim in this study.

This study has several limitations. One is rescue was self-reported by children without an ability to confirm whether a victim was truly at risk of fatal drowning. Given that almost a tenth (8.1%) continued to swim or play after being rescued, over-reporting may have occurred. However, almost three-quarters (73.7%) of rescue victims discontinued what they were doing, 2.2% of victims were unconscious and there was one fatal drowning. These numbers are consistent with real drowning risk.

Using two different samples of children is another limitation. The design does not allow a precise determination of either the 1-month or the annual rescue rates as the two surveys represent snapshots of different samples of children at two points of time, purposefully chosen to maximise the likelihood of encountering recreational play in water or swimming. Frequent, serial monitoring of a large cohort is required for that which is currently in process. Despite the inability of this study to precisely characterise rescue incidence rates, children are reporting water-based rescues of other children at substantial rates, even after allowing for potential over-reporting.

While rescue represents a potential risk, it also represents a potential newly recognised intervention for reducing early child drowning. Given the culture and the nature of present child supervision practices in rural Bangladesh, it is unlikely that it can be prevented through messages to parents regarding the need for increased supervision. Development of more effective risk knowledge and adult supervision interventions is an urgent priority. Until then, since the large majority of reported rescues are occurring as water rescues with direct contact, children should be taught safe water rescue techniques to minimise their risk and making it as safe as possible.

**What is already known on the subject**

- Drowning is a leading cause of death in children in Bangladesh.
- Children who can swim are less likely to drown.
- SwimSafe, a basic swimming and safe rescue curriculum, is effective in preventing child drowning for children when they have completed the programme.

**What this study adds**

- Children frequently report rescuing other children in rural Bangladesh. In this study, none of the reported rescues involved adult assistance to the children rescuing other children.
- All the child rescuers were older children who reported rescuing children an average of 6 years younger than them.
- Most reported rescues took place in ponds or ditches and within 10 m of the bank while the victim was bathing.
- Most reported rescues took place with the rescuer in the water and with the rescuer in contact with the victim.
